# Evaluation of Deep Learning for Caries Detection With Fine-Grained Classification and Postprocessing Improvements

**DOI:** 10.1016/j.identj.2025.100898

**Published:** 2025-07-23

**Authors:** Lin Yang, Guan-Yu Chen

**Affiliations:** a College of Electrical Engineering and New Energy, China Three Gorges University, Yichang, China; b Department of Experimental Orofacial Medicine, Philipps University Marburg, Marburg, Germany

**Keywords:** Caries detection, Intraoral photograph, Artificial intelligence, Deep learning

## Abstract

**Objectives:**

Deep learning methods have been proven to be effective in detecting dental caries in visible light images. However, existing research involves inadequate categories and mainly focuses on local lesion areas. This study aims to use advanced deep learning models to achieve caries detection based on tooth instances (where all teeth in images are detected) and fine-grained classification according to the International Caries Detection and Assessment System (ICDAS). To address the potential instability under complex scenarios, we propose 2 correction methods that incorporate background knowledge.

**Methods:**

A total of 1200 selected high-quality intraoral images were expanded to 8,754 images using data augmentation techniques, and each tooth inside was annotated. Three advanced models, YOLO-v8, YOLO-v9, and YOLO-NAS, were trained and tested on the dataset. In the stage of postprocessing, predicted categories were corrected with a weighted average of scores, and confidence scores were adaptively adjusted based on the spatial relationships of teeth.

**Results:**

The proposed methods improved the mean Average Precision (mAP) scores by 4.7% (*p* < .01/Mann-Whitney-U-test), 2.8% (*p* < .01), and 4.4% (*p* < .01) across the 3 models, with the highest score of 72.9% on YOLO-v8. Precision and recall increased by 3.8% and 5.6%, respectively, while FPS decreased from 83.1 to 78.1. Especially improved the scores for moderate caries and demonstrated greater robustness.

**Conclusion:**

The primary objectives were achieved, and the 2 proposed correction methods bring an effective improvement to the existing algorithm framework. It’s expected to promote the application of artificial intelligence and inspire further research.

**Clinical Relevance:**

This research is of clinical value due to its functional innovation: the finer classification should assist dentists formulate personalized treatment strategies. Focusing on the detailed evaluation of each tooth should help deliver better and personalised clinical care.

## Introduction

Dental caries is a worldwide oral health issue that affects human health and quality of life.^1^ If left untreated, caries can progress into pulp and periapical diseases, and may even lead to tooth loss.^2^ According to the Fourth National Oral Health Epidemiological Survey of China, the caries rate in permanent teeth among individuals aged 35-44 is 89.0%, 95.6% among those aged 55 to 64, and 98.0% among those aged 65 to 74.^3^ Similarly, reports from the United States show that approximately 90% of adults suffer from caries.^4^ However, less than 5% of global health expenditure is allocated to oral health.^5^ Due to high medical costs and inefficient manual screenings,^6^ people often struggle to understand their health status in a timely manner. When the condition worsens, dentists typically adopt conservative treatments based on the concept of minimally invasive care (MIC),^7^ leading to a higher rate of misdiagnosis (undertreatment). Additionally, early-stage caries manifests as demineralization of the enamel, which is difficult to detect with the naked eye, and traditional X-ray imaging struggles to capture small enamel lesions. Therefore, it is necessary to develop more convenient and cost-effective telemedicine technologies,^8^^,^^9^ which can be used to detect early caries lesions and assist dentists in diagnosis and decision-making.^8^

Deep learning, as a subset of machine learning, enables computer systems to automatically learn from provided data, discover patterns, and make predictions,^10^ thus can offer data-driven clinical decision support, radiographic image analysis by automation, and enhancing dental education.^11^^,^^12^ In recent years, its rapid development has made AI-based computer-aided diagnosis (AI-CAD)^13^ an important research field. In the field of dentistry, deep learning is mainly used for radiographic examinations of caries, as well as the detection of apical periodontitis and periodontitis.^14^, ^15^, ^16^, ^17^, ^18^, ^19^ However, it requires professional equipment and does not meet the needs of telemedicine. In contrast, visible light images (also known as digital images) have become a more convenient option for caries detection due to their ease of acquisition.^20^^,^^21^ At the same time, visible light images can provide detailed external features of caries and thus can serve as a supplement to X-ray images, helping dentists to analyze caries more comprehensively. In terms of image acquisition, oral endoscopes can offer high-resolution images of the inside of the mouth, allowing dentists to observe lesions and other abnormalities more precisely.^22^, ^23^, ^24^ Handheld devices such as oral cameras (OC) are also more affordable and suitable for broader use.^25^^,^^26^

In recent years, many studies have applied deep learning to visible light images. For example, Duong et al.^27^ used the support vector machine algorithm to detect caries from occlusal images of molars, which preliminarily validated the application potential of visible light images in clinical diagnosis. Convolutional Neural Network (CNN) is a typical deep learning network. By cascading multiple convolutional layers and pooling layers, it extracts features from images. This hierarchical design can handle complex patterns in images and is thus widely used. VGG19 is an early CNN architecture that only stacks multiple layers of 3 × 3 convolutions. Inception V3 processes features of different scales by parallelly combining convolutions of various kernel sizes. ResNet50 introduces skip connections to avoid the problem of gradient disappearance that is prone to occur when there are too many layers. Saini et al.^28^ compared these 3 CNN models and achieved a good accuracy of 89.98% in early caries image classification. Kühnisch et al.^21^ employed the MobileNetV2 network model to identify individual caries in oral photos, achieving over 90% accuracy. E.Y. Park^29^ first used U-Net to segment the tooth features and then applied Faster R-CNN to detect the caries lesions, they discovered that darkening background areas could improve accuracy metrics. Yoon et al.^30^ utilized the Cascade R-CNN model torecognize tooth numbers, achieving an mAP (mean Average Precision, which is the area under the relationship curve between precision and recall at different confidence thresholds) score of 0.880, and also achieved an average mAP score of 0.769 in a 3-stage caries detection task, further confirming the practicality of artificial intelligence in clinical settings.

Previous studies have made significant progress in caries detection using new technologies, but there is still room for improvement in enhancing the applicability and impact of artificial intelligence algorithms.

1. The categories of caries are relatively limited, most studies use Code 3^31^ or Code 4^32^ from the ICDAS as cutoff points, where lesions showing only more obvious caries features are classified as caries for a single-category detection task. Although some studies^33^ expanded the categories to 3 stages, if Code 0 to Code 6, including healthy teeth, are incorporated into a 7-category classification, it could further provide valuable support for dentists in formulating personalized treatment strategies.
2. Most studies focus on detecting caries in images containing a single tooth^21^ or detecting lesions in the complete oral image.^9^ In contrast, detecting and classifying all teeth in oral images aligns better with clinical examination and recording habits.

In dental practice, misdiagnosis may lead to the neglect of mild caries (active caries in ICDAS 1-3), worsening the condition. Specifically, dental caries is caused by the acidic products resulting from the fermentation of food, which lead to the dissolution of tooth minerals. If no measures are taken, bacteria and acids will penetrate the damaged tooth surface and continue to attack the deeper layers of the tooth, eventually leading to pulp infection and pulp necrosis.^34^ While overdiagnosis results in unnecessary restorative treatments, increasing patient discomfort and financial burden. Therefore, accurately recognizing caries, especially in the early stage, is crucial in this context. *This study aims to implement caries detection based on tooth instances (where the teeth in the image are captured by prediction boxes) and fine-grained classification (combining the 7 categories of ICDAS).* In preliminary experiments, we observed several critical issues that require resolution.

1. With an increase in categories, accuracy inevitably decreases. Given the correlation between ICDAS Code 0 to Code 6, it may be feasible to use this correlation to correct the predicted categories in the post-processing stage.
2. In detecting all tooth instances, factors like insufficient exposure and crowded teeth may lead to some incorrect detections. It is also possible to adaptively adjust the confidence score based on the spatial distribution features of the teeth, such as increasing or decreasing the confidence score of bounding boxes in background areas to avoid false positives.

We hypothesize that through the category and confidence corrections mentioned above, it is expected to help overcome potential issues in detection and improve detection performance.

## Materials and methods

### Data configuration

#### Dataset description

The dataset used in this study is sourced from the Kaggle Dentalai dataset,^35^ which comprises a total of 2,495 annotated oral images with widths ranging from 332 pixels to 5920 pixels. However, there are some problems with the original images. Firstly, some of the images are of low quality, possibly due to the use of different models of cameras, smartphones, and other devices, resulting in significant variations in image size and resolution. Some images not only show oral situations but also include the user's actual living environment. Additionally, these images contain varying degrees of noise. To avoid the problem of poor image quality in the original dataset, we performed data cleaning, manually filtered out low-quality images, and retained only those images with both height and width pixels greater than 1000. Ultimately, 1,200 images (about half of the original dataset) were used as the dataset for our experiments.

#### Image annotation

The annotations in the original dataset only roughly identified 2 categories: “Teeth” and “Caries.” This binary annotation hardly fully meets the need for fine-grained detection in some clinical scenarios, thereby re-annotating the tooth instances with finer classification is necessary. To minimize the ambiguity of diagnosing caries based solely on images, 6 trained medical assistants were enlisted to perform 7-category annotations according to the ICDAS,^36^ using the automatic annotation software Anylabelling (version v0.4.8; https://github.com/vietanhdev/anylabeling). The ICDAS codes range from 0 to 6, increasing with the severity of the lesions, the detailed introduction is provided below. *For brevity, this paper abbreviates “ICDAS” with “C.”*

1. C0 – healthy teeth, and the annotators need to exclude conditions such as tooth wear and discoloration of the fissures caused by non-cariogenic habits (like tea drinking).
2. C1 – visual changes in enamel, including opacity changes, white or brown lesions.
3. C2 – significant visual changes that the discolorations beyond the area of the fissure.
4. C3 – localized enamel destruction, where opacity or chalky white demineralization.
5. C4 – noticeable discoloration from the internal dentin, usually blue or brown.
6. C5 – the formation of an open cavity in the fissure, usually with demineralization.
7. C6 – the cavity affects half of the tooth surface and exposes the underlying dentin.

**Fig. 1:**
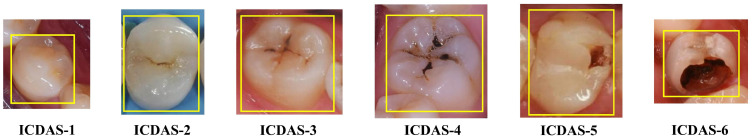
Illustration of dataset samples and annotation.

Some manually annotated ground truth information is shown in Figure 1. Each image was annotated independently by at least 2 annotators. In case of disagreement, the annotations should be flagged and referred to dental therapists for more reliable annotations. After completing this series of work, 2 evaluators reviewed and appropriately corrected the annotation results. The final Kappa coefficient was calculated to be between 0.7 and 0.9, indicating the achievement of substantial consistency.

#### Data augmentation

**Fig. 2:**
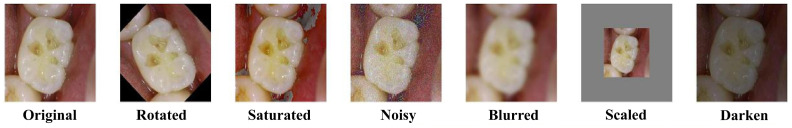
Illustration of used augmentation methods.

For deep learning tasks, small datasets are prone to overfitting, which limits the generalizability of the network. To address this, we employed data augmentation techniques to expand the dataset. Specifically, images were rotated by 45° with a 40% probability, with 50% of these rotations being clockwise and 50% counterclockwise. The brightness and contrast were adjusted with a 30% probability, noise and blurring were added with a 25% probability, and scaling by a factor of 2 was applied with a 20% probability. In the end, 8754 images, including the original ones, were obtained, as shown in Figure 2. These random transformations help to more comprehensively simulate possible real-world scenarios, improving the robustness of the model.

#### Dataset partition

Table 1 shows the statistics of caries instances in each category in the dataset. It can be seen that mild instances account for the largest proportion, while severe instances are less common, presenting an overall long-tail distribution. To ensure a sufficiently large training set, a validation set to adaptively adjust the training strategy, and a reliable test set to evaluate model performance, we divided the data into training, validation, and test sets in an 8:1:1 ratio, with 7004 images in the training set, 875 images in the validation set, and 875 images in the test set. These 3 sets are independent, meaning that the same image and all of its augmented images appear in only one set (that is, the unaugmented original images are randomly assigned to each subset according to the predetermined ratio, and then their augmented images are added to each respective subset).

**Table 1:** Statistics of caries instances in each category.

Dataset	C0	C1	C2	C3	C4	C5	C6	Photos
Total	9443	21,102	14,502	8531	6706	725	271	8754
Training	7349	16,637	11,640	7114	5321	575	211	7004
Validation	983	2150	1394	813	653	75	28	875
Test	1111	2315	1468	604	732	75	32	875

### Proposed methods

#### Non-maximum suppression

Most of the network models^37^^,^^38^ divide a single image into n × n sub-regions, generating corresponding predictions at the anchor points based on local features, as shown in the lower right corner of Figure 5, here *80*^*2*^*+40*^*2*^*+20*^*2*^ refers to the number of anchor points at the 3 feature layers of the network. The number *4* represents the information about the center coordinates and width and height of the predicted bounding boxes. *n_cls* refers to the number of categories involved in the prediction, which is 7 in this paper. However, a target often occupies multiple sub-regions, resulting in multiple redundant predictions. Non-Maximum Suppression (NMS) is a necessary step to remove these redundant boxes. Specifically, it includes:

1. Selecting the maximum score across all categories at each anchor point as the prediction score, and the corresponding category as the predicted class.
2. Comparing the prediction scores at each anchor point with a set confidence threshold, and then eliminating low-confidence predictions.
3. Among the remaining predictions, calculating the Intersection over Union (IoU) between the highest-scoring box and other boxes. If the IoU is greater than the set threshold, it indicates that the 2 boxes are likely to correspond to the same target, and thus the one with the lower score is removed.

As a greedy algorithm focused on local optimization, it is continuously improved. Soft-NMS^39^ replaces the traditional IoU-based suppression criterion by weighting the confidence with 1−IoU. IoU-Net^40^ and Softer-NMS^41^ introduce an IoU branch and a variance branch into the detection head, respectively, to separately represent the uncertainty of the location regression results to address the issue of spatial misalignment.^42^ DB-NMS^43^ applies a Density Peak Clustering (DPC) algorithm^44^ to filter redundant boxes based on geometric similarity, enhancing the ability to distinguish different objects.

#### Category correction

**Fig. 3:**
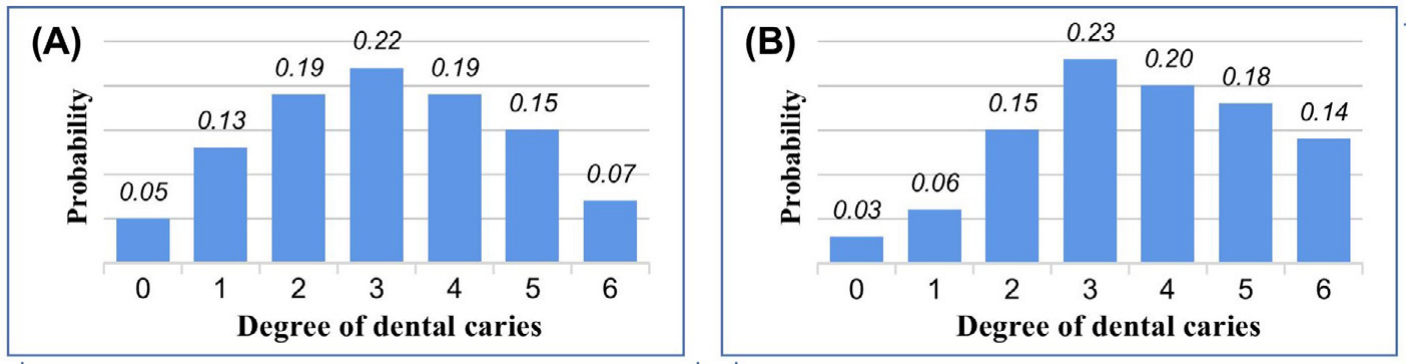
Illustration of category correction.

In the ICDAS's 7 categories, there is a certain visual similarity between adjacent categories.^45^ Specifically, if the expert classification of a tooth is C3, the neural network model's predicted scores for nearby categories (C2, C4) will be significantly higher than those for distant categories (C0, C6). From a statistical perspective, the overall scores follow a normal distribution with a peak at the correct class, as shown in Figure 3A.

The traditional post-processing algorithm selects only the category with the highest score as the prediction category:

$$Class_{prediction}=argmax_{class}\left(score_{class}\right)$$

The overall distribution is neglected in this approach. Sometimes, the network model's predicted scores follow a skewed distribution, as shown in Figure 3B. Although the score under C3 is still the highest, overall, the scores on its right side are much larger than those on the left, making C4 closer to the truth category. To fully consider the scores across all categories, it's needed to explicitly introduce this background knowledge. The intuitive idea is to average the 7 categories with their corresponding scores as weights and then round to get the corrected category.

$$Class_{prediction}=round\left[\frac{\sum_{class=0}^{6}class\cdot score_{class}}{\sum_{class=0}^{6}score_{class}}\right]$$

However, there is a new shortcoming arising: when the expert classification falls into edge categories C0 or C6, the calculated weighted average is then constantly greater than 0 or less than 6. This means that the weighted average calculation will guide the output of edge categories toward the middle categories. To prevent this, we cancel this weighting when the original predicted category is C0 or C6 and revert to the greedy output.

#### Confidence correction

**Fig. 4:**
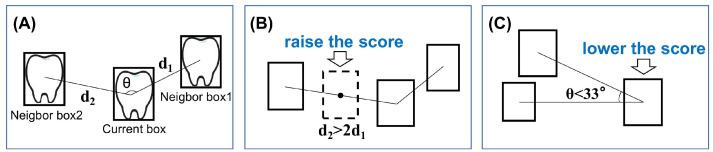
Illustration of confidence correction.

In a well-aligned dentition, adjacent teeth typically form a nearly equidistant linear distribution, as shown in Figure 4A. If a round of NMS with a higher confidence threshold (0.5) is first performed, for the retained single high-confidence box, the 2 nearest high-confidence boxes will be located on either side, and the 2 distances will be similar. This relationship can be quantified as: 1. d2/d1 < 1.5; 2. θ > 90°.

If d2/d1 > 2 (Figure 4B), it indicates a potential false negative (FN) between the current box and the second neighbor box, corresponding to teeth with less prominent features that were not assigned a high score by the network, such as wisdom teeth in the corners or crowded canines. Therefore, we can simulate this FN, as shown by the dashed box in Figure 4B, and the low-confidence boxes with an IoU greater than 0.6 will be the boxes where score enhancement is needed. After experimenting with many options, it has been proven that their scores are best corrected using the following formula ((1-score) is the normalization term to ensure that the enhanced score does not exceed 1.0):

score←score+0.5×(1-score)

If θ < 33° (Figure 4C), the current box is more likely located in the background area, far from other teeth, but is assigned a higher score due to the presence of features similar to teeth, such as a mouth opener or bubbles on the lens. In this case, the false positive (FP) score is suppressed by multiplying a small factor. If d2/d1 > 2.7, it indicates that the current box is located at the edge. As with the previous formula, the score will be increased to eliminate the FN.

After adjusting the scores, NMS is performed again with the original threshold. Since FP and FN are determined based on the quantitative relationship between confidence and threshold, it is practical to adjust the confidence score for boxes in potential regions by using the characteristics of tooth distribution as motivation. The constants in the conditions d2/d1>2 and θ<33° are empirical values obtained through tuning. In classic algorithms Faster R-CNN and SSD, we tested d2/d1 at intervals of 0.5 between 1 and 4, and θ was tested at intervals of 5° between 10° and 40°. It was found that the maximum mAP scores were achieved near 2 and 33°, respectively. To ensure the experiment's objectivity, this hyperparameter setting has been extended to the latest YOLO algorithms.

#### Baseline deep learning algorithms

In the field of 2D object detection, the sensitivity and specificity of the YOLO model are significantly superior to other detection algorithms.^9^ This study conducts experiments using its latest state-of-the-art (SOTA) versions YOLO-v8,^46^ YOLO-v9,^47^ and YOLO-NAS,^48^ to fully verify the generalizability of the proposed methods.

**Fig. 5:**
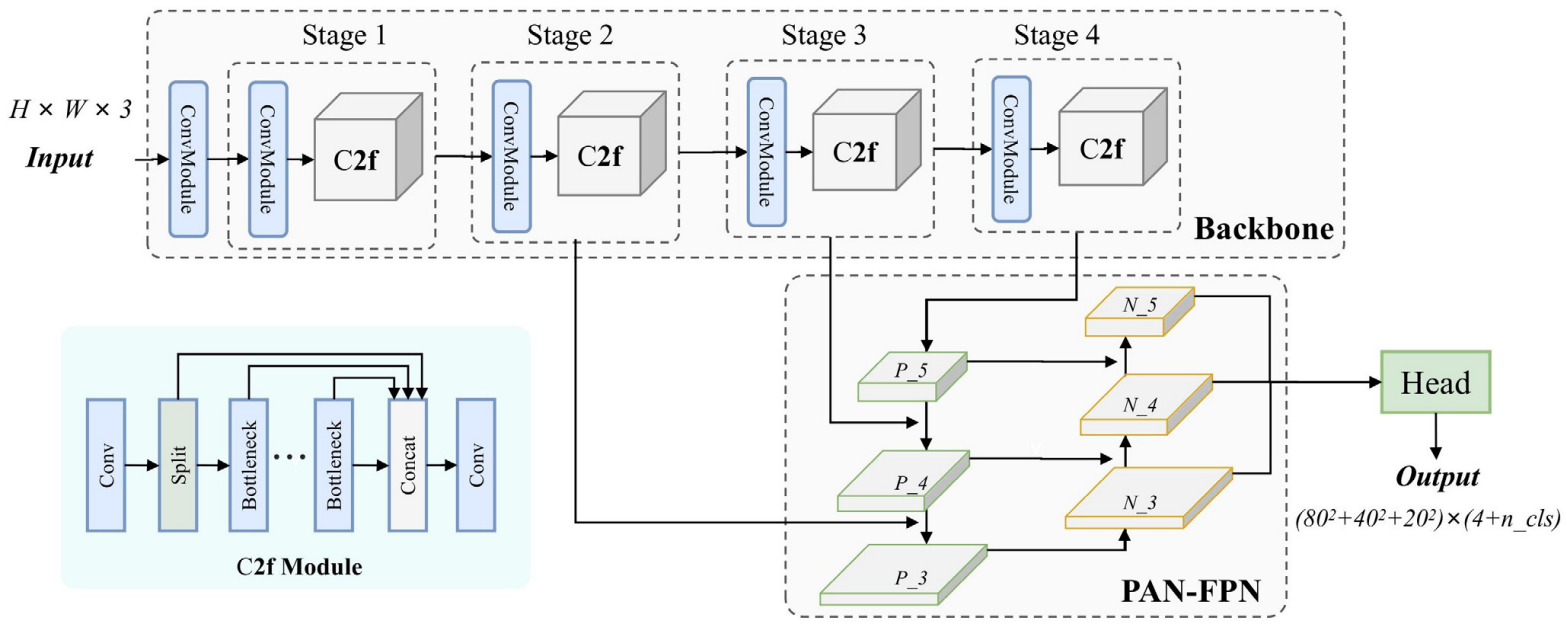
The architecture of the YOLOv8.

The YOLO-v8 model structure is shown in Figure 5 and consists of 3 parts. The backbone network is responsible for extracting features from the input image, with the semantic information becoming more complex and abstract in the later stages. Its core module is C2f, which uses gradient diversion technology to reduce computation while increasing network depth. The neck network is used to fuse the feature information from the last 3 stages, consisting of the top-down FPN and bottom-up fused PAN. The head network uses decoupled detection heads with shared feature maps for specific classification and location regression predictions.

In the 2 versions presented later, YOLO-v9 combines the advantages of CSPNet and ELAN to form the GELAN architecture, balancing lightweight design, inference speed, and accuracy. It introduces Programmable Gradient Information (PGI) to address the issue of information loss and network updates during the feature extraction process. YOLO-NAS designs quantization-aware modules named QSP and QCI to minimize accuracy loss during quantization, and the AutoNAC automatic architecture adapts the algorithm for user needs, particularly suitable for real-time edge devices.

### Experimental settings

The model training and testing were conducted using the PyTorch framework (version 1.10.1; https://pytorch.org/) on the Ubuntu 16.05 operating system of the AutoDL cloud server rental platform, equipped with an NVIDIA TITAN RTX 2080 Ti GPU, Xeon(R) Platinum 8255C 2.50 GHz CPU, and 32 GB RAM (2 × 16 GB) DDR4 @ 3600 MHz memory. The Python version was 3.8, and the CUDA version was 11.3. The initial learning rate of the model was set to 0.01, with weight decay of 0.0005, momentum set to 0.807, and the Stochastic Gradient Descent optimizer used to update model parameters. The random seed was set to 11. In the first 50 epochs, the backbone network weights were frozen, and the training was performed with a batch size of 2. From epoch 51, the model was unfrozen and trained with a batch size of 4, iterating for a total of 400 epochs. The input image size was 640 × 640.

During post-processing, the confidence threshold was set to 0.1, and the IoU threshold for NMS was set to 0.5. We used common evaluation metrics such as precision, recall, mAP score, and FPS to assess the algorithm's performance on the test set. By evaluating precision and recall at different confidence thresholds, we can plot the R-P curve. When the IoU threshold between the predicted box and the ground truth is 50%, the area under the curve and the axes defines the mAP@50, which is more suitable for the multi-class and localization tasks in this study than the F1 score.

## Results

The results of the comparison and ablation experiments are shown in Table 2. Among the 3 baseline algorithms, YOLO-v8 achieved the highest mAP score of 0.682, YOLO-NAS demonstrated a similar performance of 0.677, and although YOLO-v9 had lower performances in accuracy metrics, it had the fastest inference speed of 92.81 FPS. The combination of proposed correction methods improved the mAP score by 4.7%, 2.8%, and 4.4%, respectively. Specifically, category correction mainly affected precision (+2.7%, +0.9%, +2.6%), while confidence correction significantly improved recall (+3.6%, +2.3%, +3.5%), but there was a slight loss in computational efficiency.

**Table 2:** Test results with or without proposed 2 corretions.

Model	Correction		Precision	Recall	mAP@50 (95% CI)	*P*-value (if significant)	FPS
	Cls	Conf					
YOLO-v8	No	No	0.592	0.678	0.682(0.667-0.718)	**—**	**83.12**
	Yes	No	0.619	0.715	0.706(0.685-0.727)	.0235(√)	81.36
	No	Yes	0.603	0.714	0.721(0.712-0.753)	.0054(√)	72.17
	Yes	Yes	**0.630**	**0.734**	**0.729**(0.709-0.750)	**.0031**(√)	78.07
YOLO-v9	No	No	0.570	0.638	0.625(0.611-0.647)	**—**	92.81
	Yes	No	0.579	0.644	0.642(0.631-0.648)	.0549(×)	**92.93**
	No	Yes	0.570	0.661	0.649(0.632-0.661)	.0062(√)	91.01
	Yes	Yes	**0.593**	**0.666**	**0.653**(0.641-0.670)	**.0010**(√)	89.47
YOLO-NAS	No	No	0.552	0.676	0.677(0.650-0.792)	**—**	**86.41**
	Yes	No	0.578	0.690	0.717(0.701-0.721)	.0093(√)	83.74
	No	Yes	0.563	0.711	0.708(0.694-0.721)	.0029(√)	74.12
	Yes	Yes	**0.602**	**0.729**	**0.721**(0.704-0.749)	**.0004**(√)	71.60

*Note.* The bold represents that the model has the best performance under this indicator.

Moreover, to verify whether the proposed methods bring statistically significant improvements to the algorithm's performance, we trained 3 baselines with 20 different random seeds. The Shapiro-Wilk test (*p* >.1) confirmed that the resulting mAP scores do not follow a normal distribution. We then performed a Wilcoxon non-parametric test on the mAP score sets of the improved algorithm and the baseline. The resulting *p*-values are included in Table 2, showing that our correction method has general significance (*p* < .05). The 1.7% improvement brought by the category correction applying solely to the YOLO-v9 shows no significant difference from the baseline. However, the combination of correction methods showed the best significance in all algorithms (*p* < .01).

The detailed diagnostic performance is shown in Table 3. In the original YOLO-v8, the recall rate is generally higher than the precision, which is related to the similarity of visual features between adjacent categories. Additionally, predictions with lower confidence score often have higher classification uncertainty, further lowering precision. Regarding precision, there are 2 peaks at C2 and C6. The category correction excels in intermediate categories such as C2, C3, and C4, while the confidence correction is concentrated on C4 and C5. The recall rate initially decreases with the severity of caries and then increases again at C5 and C6. The impact of category correction remains similar to that on precision, while confidence correction brings at least a 2% improvement across all categories. The overall improvement is more striking in recall.

**Table 3:** Diagnostic performance metrics for all ICDAS categories.

Class	Precision				Class	Recall			
	Original	Cls-cor	Conf-cor	Ours		Original	Cls-cor	Conf-cor	Ours
C0	55.3	55.6	55.8	56.2	C0	**78.1**	**78.9**	**80.6**	**80.6**
C1	61.5	64.0	62.7	64.9	C1	73.3	76.4	75.0	77.6
C2	**63.5**	**67.8**	**63.9**	**68.9**	C2	66.4	71.0	69.6	72.1
C3	59.4	63.9	59.8	65.0	C3	62.7	67.9	66.6	69.3
C4	54.2	57.1	56.8	58.9	C4	58.0	64.1	64.2	66.6
C5	57.6	61.1	59.4	62.6	C5	65.1	69.7	69.5	72.0
C6	63.3	63.5	63.5	64.2	C6	71.0	72.4	74.3	75.7
Avg	59.3	61.9	60.3	63.0	Avg	67.8	71.5	71.4	73.4

*Note.* The bold represents that the model has the best performance under this indicator.

**Fig. 6:**
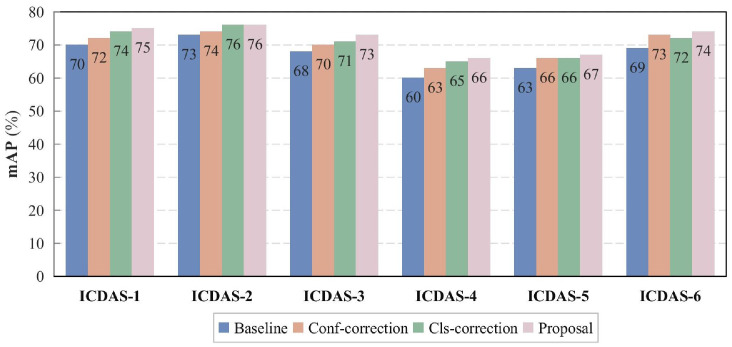
mAP scores of the algorithm in different stages of dental caries.

Figure 6 visually displays the impact of the proposed methods on the mAP scores across different categories of caries in the form of histograms. It can be observed that in the original YOLO-v8 algorithm, the precision metrics for moderate caries C3 and C4 were the lowest, and category correction significantly improved classification performance at this stage by 5.3% and 6.0%, respectively. Confidence correction mainly optimized the scores for moderate and severe stages. The improvements in the mild stages C1 and C2 were relatively minor, likely because the closer proximity of the anterior incisors, with lower caries incidence and better exposure, allowing the neural network model to generate more accurate predictions, thus requiring fewer corrections.

**Fig. 7:**
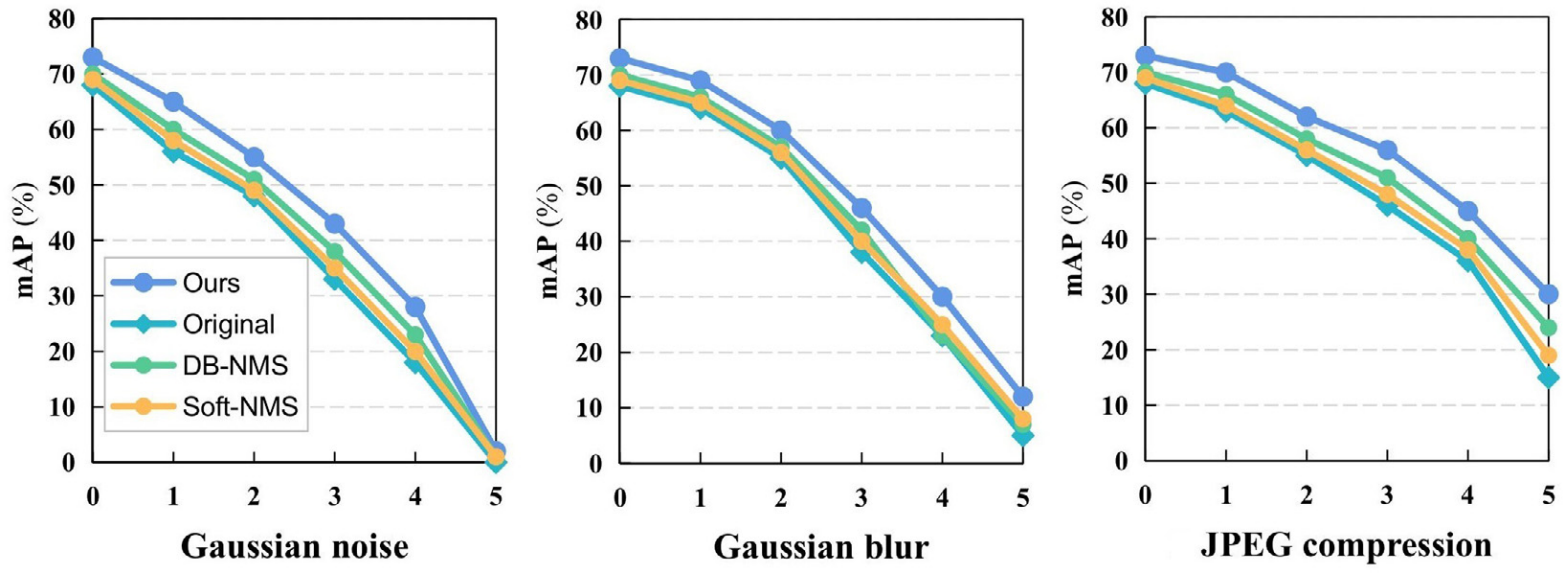
Performance comparison results after different degree of image distortion.

The detection capability of the algorithm may encounter various influences in real-world environments, such as noise in the captured images or compression caused during file transmission. Therefore, it is crucial to evaluate the algorithm's robustness. Previous studies^49^ applied up to 15 image distortion assessment models to evaluate performance. This study selects 3 representative types of distortions (Gaussian noise, Gaussian blur, and JPEG compression) and compares our correction methods with other competitive post-processing methods, Soft-NMS and DB-NMS, as shown in Figure 7. It is clear that after postprocessing correction, the performance of the corrected algorithm consistently outperforms other algorithms, fully demonstrating that our method is more robust.

**Fig. 8:**
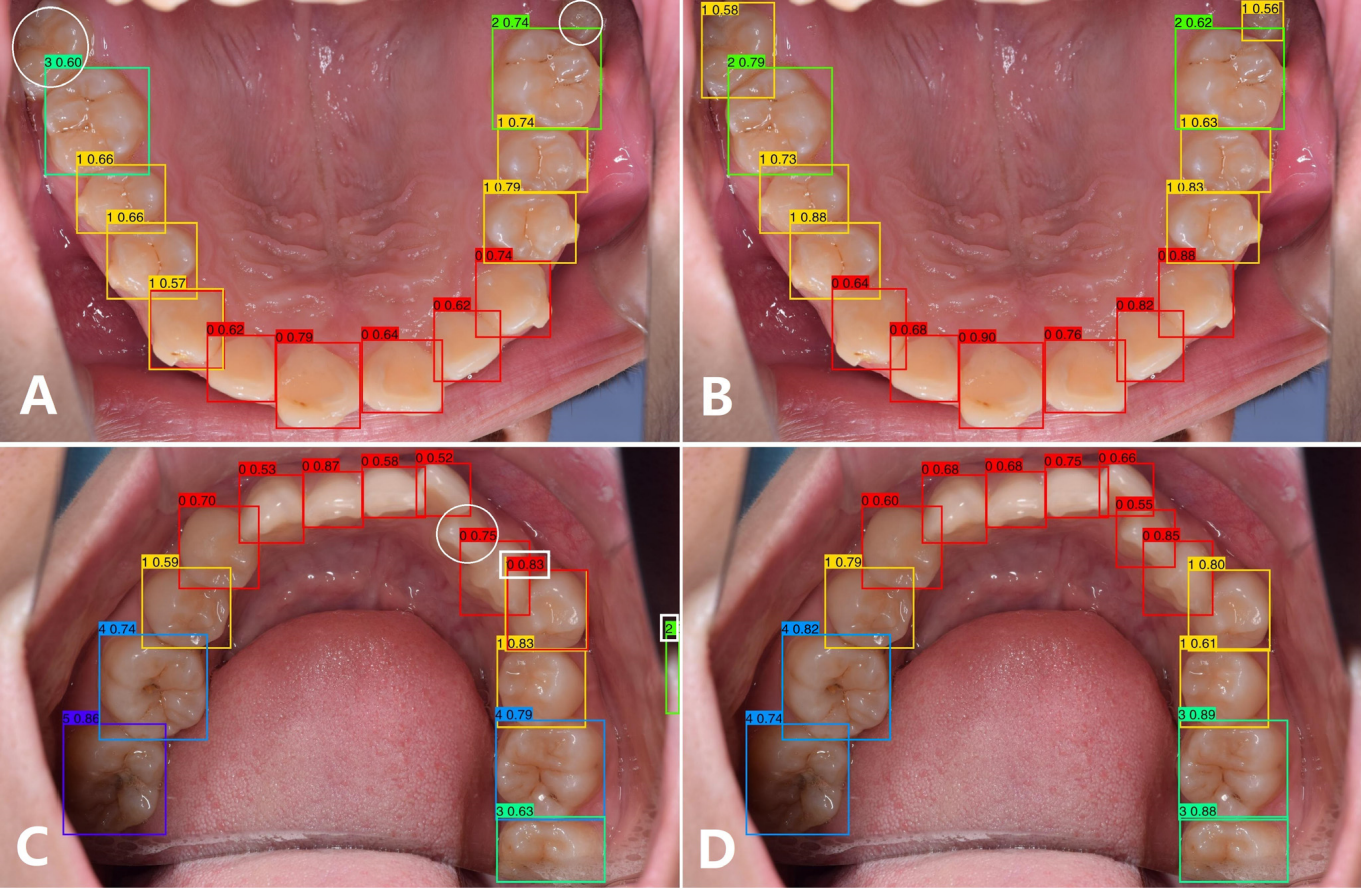
Visualization of original caries detection results and the improved output with our methods.

To clearly exhibit the results of the algorithm before and after improvements in tooth instance detection and ICDAS fine-grained classification, 2 intraoral photos were selected for visualization, as shown in Figure 8. The left images show the baseline prediction, and the right images show the improved prediction. The numbers in the top-left corner of the prediction boxes represent the ICDAS code and the probability value, respectively. From Figures 8A and C reveal some shortcomings in the original detection results. In the white circle area, 3 FN cases occurred due to the remote and crowded location, and in the white rectangle area, 4 FP cases were caused by ambiguous features. These errors were satisfactorily resolved in Figures 8B and D, where the integration of background knowledge endowed the algorithm with stronger reasoning capability. It is worth noting that the molar in the upper-right corner of Figure 8B is classified as C1. Although this is technically correct, since it is a nonactive lesion, it may lead to cases of overdiagnosis. Therefore, appropriate human intervention is still essential during clinical application.

## Discussion

This study focuses on improving fine-grained caries detection in intraoral photos. By introducing 2 simple yet effective post-processing correction methods, the mAP scores of the original 3 models—YOLO-v8, YOLO-v9, and YOLO-NAS—were increased by 4.7%, 2.8%, and 4.4%, respectively. The maximum mAP score of 72.9% was achieved with YOLO-v8, particularly improving the performance of moderate caries, which previously showed the lowest scores. The algorithm also demonstrated better robustness compared to other enhanced methods. The visualizations of the results further confirmed the effectiveness of the improvements. One of the highlights is that detection and classification are performed simultaneously, which largely shortens the diagnostic process. Overall, our work shows promising results and helps promote the application of visible light images in clinical caries diagnoses.

In terms of lesion detection, the model's highest sensitivity of 73.4% is higher than the 64.6% reported in a previous study using smartphone-captured images.^50^ Both the mAP score of 72.9% and the precision of 63.0% are also higher than those in another study combining Transformer mechanisms,^51^ which reported 56.9% and 62.3%, respectively. The improvement in the YOLO algorithm, with increases of 4.7% and 4.4%, surpasses the 2.1% and 3.0% improvements shown in a study using tiling strategies,^52^ demonstrating stronger performance. In the case of caries classification at different stages, our results, with an mAP score of 68.2% at baseline, are comparable to the 71.8% and 69.5% reported in a previous study using standardized intraoral photos.^30^ The inclusion of the improved algorithm has brought about a new boost in performance, effectively enhancing the reliability of automated diagnostic results, and highlighting the necessity of correcting predictions.

Some interesting phenomena can also be observed from the results. The number of instances for C6 is much smaller than for C4 and C5, but the detection performance is better. We believe that, in severe cases, the caries has already exposed the underlying dentin, making its visual features easier to distinguish. The number of instances for C1 is greater than that for C2, but its precision is lower. This anomaly may be due to the subtle differences between mild caries and healthy teeth.^9^ Furthermore, multiple factors can interfere with diagnostic results. External factors include insufficient exposure, imaging instability, and fog on the mirror, which can be addressed by image enhancement techniques simulating real-world blurriness and noise.^53^ Internal factors include other clinical symptoms such as dental calculus, white spot lesions, and surface pigmentation. Therefore, during the data cleaning phase, we only retained data on healthy teeth and caries at different stages of lesions to help the model focus on task-specific features. Another approach is to incorporate these factors into the algorithm; and multi-task learning^54^^,^^55^ may also help improve result accuracy.

Although the use of deep learning-based automated prediction tools can improve diagnostic efficiency, the supervision of clinicians remains indispensable in this process. In addition to the clinician's intuition and extensive experience, which can effectively rule out the interfering factors discussed above, external factors such as the patient's age, medical history, and dietary habits are also crucial considerations for evaluation and the formulation of personalized treatment plans. For borderline or ambiguous lesions, clinicians need to confirm the diagnosis using a broader range of methods, such as X-rays and probing, which emphasizes the importance of expert supervision.

There are also some limitations in our work, here we discuss them and propose possible solutions for future research.

1. Compared to X-ray images, intraoral images cannot express the internal information of teeth. Therefore, there are limitations in fundamentally detecting all caries. Imaging data should be used as a supplement to improve diagnostic results, or multiple photos of caries should be taken from different angles, and the network model can then integrate predictions from various perspectives.
2. The current visible light datasets, especially open-source datasets, are relatively limited. This requires cooperation and resource sharing among multiple medical institutions. Although data augmentation can improve the model's robustness, it cannot provide new features. A dataset covering more caries cases will provide important support for the algorithm's progress.
3. The reference information provided by annotators directly impacts the reliability of the model. Therefore, including more annotators and inviting experienced experts for guidance will undoubtedly have a more positive impact on the real-world application of artificial intelligence methods.

The proposed methods have been validated in relatively idealized experiments. It has been noticed that the confidence correction slightly reduces the algorithm's real-time performance, but with an FPS of 78.1, in absolute terms, it is relatively high compared to manual screening, thus still acceptable in most clinical scenarios, and as hardware facilities improve, this issue will also be weakened. In future research, from a technical perspective, we plan to further optimize the algorithm to overcome the computational overhead caused by the confidence correction, enhancing the real-time performance and scalability of the detection algorithm. From an application perspective, we aim to deploy the model algorithm onto various mobile devices or embedded platforms for feasibility and impact evaluation, and explore ways to ensure patient privacy and security, thereby more widely improving human oral health.

## Conclusion

The clinical diagnosis of caries plays an important role in maintaining human health. This study introduces new artificial intelligence technologies into caries detection to provide more efficient and low-cost solutions. Using advanced YOLO deep learning models, namely YOLO-v8, YOLO-v9, and YOLO-NAS, we have achieved caries detection based on tooth instances and fine-grained classification, with preliminary results. To improve model performance, 2 postprocessing correction methods are proposed: category correction, which comprehensively considers score distribution to integrate into more reliable output, and confidence correction, which adjusts scores based on the spatial distribution pattern of instances to capture more accurate tooth instances. This study better aligns with the requirements of clinical caries detection, promotes the application of visible images and artificial intelligence, and is expected to realize patient self-examination and computer-aided diagnosis.

## Ethical approval

Ethical approval and consent were not required as this study was based on publicly available data (retrieved at https://www.kaggle.com/datasets/pawanvalluri/dental-segmentation).

## CRediT authorship contribution statement

**Lin Yang:** Conceptualization, Data curation, Formal analysis, Methodology, Validation, Writing – original draft, Writing – review & editing. **Guan-Yu Chen:** Supervision, Writing – original draft, Writing – review & editing.

## Conflict of interest

The authors declare that they have no known competing financial interests or personal relationships that could have appeared to influence the work reported in this paper.

## Appendix Supplementary materials

Image, application 1

## References

[1] Wang C., Zhang R., Wei X., Wang L., Xu W., Yao Q. (2022). Machine learning-based automatic identification and diagnosis of dental caries and calculus using hyperspectral fluorescence imaging. Photodiagnosis Photodyn. Ther..

[2] Wolf T.G., Cagetti M.G., Fisher J.-M., Seeberger G.K., Campus G. (2021). Non-communicable diseases and oral health: an overview. Front Oral Health.

[3] Wang X. (2018).

[4] Heng C.C. (2016). Tooth decay is the most prevalent disease. Federal Practitioner.

[5] Piran M., Sharifi A., Safari M. (2023). Exploring the roles of education, renewable energy, and global warming on health expenditures. Sustainability.

[6] Baelum V., Hintze H., Wenzel A., Danielsen B., Nyvad B. (2011). Implications of caries diagnostic strategies for clinical management decisions. Commun Dent Oral Epidemiol.

[7] Zohar I., Ben David D., Schwartz O., Pomerantz A., Caliari G., Hoffman E. (2024). Amikacin treatment in patients with Enterobacterales bacteraemia: impact of MIC on mortality. J Antimicrob Chemother.

[8] Al-Jallad N., Ly-Mapes O., Hao P., Ruan J., Ramesh A., Luo J. (2022). Artificial intelligence-powered smartphone application, AICaries, improves at-home dental caries screening in children: Moderated and unmoderated usability test. PLOS Digital Health.

[9] Thanh M., Van Toan N., Vo Truong Nhu N., Nguyen T., Cu Nguyen G., Nguyen D. (2022). Deep learning application in dental caries detection using intraoral photos taken by smartphones. Appl Sci.

[10] Zhao L., Li S. (2020). Object detection algorithm based on improved YOLOv3. Electronics.

[11] Samaranayake L., Tuygunov N., Schwendicke F., Osathanon T., Khurshid Z., Boymuradov S.A. (2025). The transformative role of artificial intelligence in dentistry: a comprehensive overview. Part 1: fundamentals of AI, and its contemporary applications in dentistry. Int Dent J.

[12] Tuygunov N., Samaranayake L., Khurshid Z., Rewthamrongsris P., Schwendicke F., Osathanon T. (2025). The transformative role of artificial intelligence in dentistry: a comprehensive overview part 2: the promise and perils, and the international dental federation communique. Int Dent J.

[13] Yoon J.H., Han K., Suh H., Youk J.H., Lee S., Kim E.-K. (2023). Artificial intelligence-based computer-assisted detection/diagnosis (AI-CAD) for screening mammography: Outcomes of AI-CAD in the mammographic interpretation workflow. Eur J Radiol Open.

[14] Khanagar S.B., Al-Ehaideb A., Maganur P.C., Vishwanathaiah S., Patil S., Baeshen H.A. (2021). Developments, application, and performance of artificial intelligence in dentistry – a systematic review. J Dent Sci.

[15] Lee J.-H., Kim D.-H., Jeong S.-N., Choi S.-H. (2018). Detection and diagnosis of dental caries using a deep learning-based convolutional neural network algorithm. J. Dent..

[16] Leo L., Reddy T. (2020). Dental caries classification system using deep learning based convolutional neural network. J Comput Theor Nanosci..

[17] Holtkamp A., Elhennawy K., Cejudo Grano de Oro J.E., Krois J., Paris S., Schwendicke F. (2021). Generalizability of deep learning models for caries detection in near-infrared light transillumination images. J Clin Med.

[18] Schwendicke F., Elhennawy K., Paris S., Friebertshäuser P., Krois J. (2019). Deep learning for caries lesion detection in near-infrared light transillumination images: a pilot study. J. Dent..

[19] Salehi H.S., Barchini M., Mahdian M. (2020). Optimization methods for deep neural networks classifying OCT images to detect dental caries.

[20] Bhattacharjee N. (2022). Automated dental cavity detection system using deep learning and explainable AI, AMIA ... Annual Symposium proceedings. AMIA Symposium.

[21] Kühnisch J., Meyer O., Hesenius M., Hickel R., Gruhn V. (2021). Caries detection on intraoral images using artificial intelligence. J. Dent. Res..

[22] Vinayahalingam S., Kempers S., Schoep J., Hsu T.H., Moin D.A., van Ginneken B. (2023). Intra-oral scan segmentation using deep learning. BMC Oral Health.

[23] Albano D., Galiano V., Basile M., Di Luca F., Gitto S., Messina C. (2024). Artificial intelligence for radiographic imaging detection of caries lesions: a systematic review. BMC Oral Health.

[24] Li X., Zhao D., Xie J., Wen H., Liu C., Li Y. (2023). Deep learning for classifying the stages of periodontitis on dental images: a systematic review and meta-analysis. BMC Oral Health.

[25] Pentapati K., Siddiq H. (2019). Clinical applications of intraoral camera to increase patient compliance - current perspectives. Clin Cosmetic Investig Dent.

[26] Aly N.M., Kashlan M., Giraudeau N., El Tantawi M. (2024). Comparison of intraoral cameras and smartphones in early childhood caries detection: a diagnostic accuracy study. J Evid Based Dent Pract.

[27] Duong D.L., Kabir M., Kuo R. (2021). Automated caries detection with smartphone color photography using machine learning. Health Informatics J.

[28] Saini D., Jain R., Thakur A. (2021). Dental Caries early detection using Convolutional Neural Network for Tele dentistry. 2021.

[29] Park E.Y., Cho H., Kang S., Jeong S., Kim E.-K. (2022). Caries detection with tooth surface segmentation on intraoral photographic images using deep learning. BMC Oral Health.

[30] Yoon K., Jeong H.-M., Kim J., Park J.-H., Choi J. (2023). AI-based dental caries and tooth number detection in intraoral photos: model development and performance evaluation. J. Dent..

[31] Park E., Cho H., Kang S., Jeong S., Kim E.-K. (2022). Caries detection with tooth surface segmentation on intraoral photographic images using deep learning. BMC Oral Health.

[32] Kang S., Shon B., Park E.Y., Jeong S., Kim E.K. (2024). Diagnostic accuracy of dental caries detection using ensemble techniques in deep learning with intraoral camera images. PLoS One.

[33] Jiang H., Zhang P., Che C., Jin B., Zhu Y. (2023). CariesFG: A fine-grained RGB image classification framework with attention mechanism for dental caries. Eng Appl Artificial Intelligence.

[34] Pretty I., Ekstrand K. (2015). Detection and monitoring of early caries lesions: a review. Eur Arch Paediatr Dent.

[35] Valluri P. (2023). Dentalai Computer Vision Project. https://www.kaggle.com/datasets/pawanvalluri/dental-segmentation.

[36] Ismail A.I., Sohn W., Tellez Merchan M., Amaya A., Sen A., Hasson H. (2007). The International Caries Detection and Assessment System (ICDAS): an integrated system for measuring dental caries. Commun Dent Oral Epidemiol..

[37] Liu Z., Zheng T., Xu G., Yang Z., Liu H., Cai D. (2020). Proceedings of the AAAI Conference on Artificial Intelligence.

[38] Qiao S., Chen L.C., Yuille A. (2021). 2021 IEEE/CVF Conference on Computer Vision and Pattern Recognition (CVPR).

[39] Bodla N., Singh B., Chellappa R., Davis L.S. (2017). 2017 IEEE International Conference on Computer Vision (ICCV).

[40] Jiang B., Luo R., Mao J., Xiao T., Jiang Y., Ferrari V., Hebert M., Sminchisescu C., Weiss Y. (2018). Computer Vision – ECCV 2018.

[41] Wang Y., Rao Y., Huang C., Yang Y., Huang Y., He Q. (2021). 2021 4th International Conference on Pattern Recognition and Artificial Intelligence (PRAI).

[42] Song G., Liu Y., Wang X. (2020). 2020 IEEE/CVF Conference on Computer Vision and Pattern Recognition (CVPR).

[43] Rui L., Tang X.-S., Hao K. (2022). DB-NMS: improving non-maximum suppression with density-based clustering. Neural Comp Appl.

[44] Alex R., Alessandro L. (2014). Clustering by fast search and find of density peaks. Science.

[45] K. Kobs, M. Steininger, A. Zehe, F. Lautenschlager, A. Hotho, SimLoss: class similarities in cross entropy, 2020, pp. 431-439.

[46] Terven J., Córdova-Esparza D.-M., Romero-González J.-A. (2023). A comprehensive review of YOLO architectures in computer vision: from YOLOv1 to YOLOv8 and YOLO-NAS. Mach Learn Knowl Extr.

[47] Wang C.Y., Yeh I.H., Liao H.Y.M. (2024). Yolov9: learning what you want to learn using programmable gradient information. Eur Conf Comput Vis.

[48] Маратулы А., Абибуллаев Е.А. (2024). Performance study and comparative analysis of yolo-nas and previous versions of YOLO. Int J Inform Commun Tec.

[49] Michaelis C., Mitzkus B., Geirhos R., Rusak E., Bringmann O., Ecker A.S., et al. Benchmarking robustness in object detection: autonomous driving when winter is coming. arXiv Preprint. 2019; arXiv:1907.07484.

[50] Zhang X., Liang Y., Li W., Liu C., Gu D., Sun W., Miao L. (2022). Development and evaluation of deep learning for screening dental caries from oral photographs. Oral Dis.

[51] Jiang H., Zhang P., Che C., Jin B. (2021). RDFNet: a fast caries detection method incorporating transformer mechanism. Comput Math Methods Med..

[52] Jiang C., Zhai S., Song H., Ma Y., Fan Y., Fang Y. (2024). Object detection for caries or pit and fissure sealing requirement in children’s first permanent molars. Comput Intelligence.

[53] Ding B., Zhang Z., Liang Y., Wang W., Hao S., Meng Z. (2021). Detection of dental caries in oral photographs taken by mobile phones based on the YOLOv3 algorithm. Ann Transl Med.

[54] Xiong Y., Zhang H., Zhou S., Lu M., Huang J., Huang Q. (2024). Simultaneous detection of dental caries and fissure sealant in intraoral photos by deep learning: a pilot study. BMC Oral Health.

[55] Liu Y., Cheng Y., Song Y., Cai D., Zhang N. (2024). Oral screening of dental calculus, gingivitis and dental caries through segmentation on intraoral photographic images using deep learning. BMC Oral Health.

